# Simultaneous Maximum-Likelihood Reconstruction of Absorption Coefficient, Refractive Index and Dark-Field Scattering Coefficient in X-Ray Talbot-Lau Tomography

**DOI:** 10.1371/journal.pone.0163016

**Published:** 2016-10-03

**Authors:** André Ritter, Gisela Anton, Thomas Weber

**Affiliations:** Friedrich-Alexander-Universität Erlangen-Nürnberg (FAU), Erlangen Centre for Astroparticle Physics (ECAP), Erwin-Rommel-Str. 1, 91052 Erlangen, Germany; Chongqing University, CHINA

## Abstract

A maximum-likelihood reconstruction technique for X-ray Talbot-Lau tomography is presented. This technique allows the iterative simultaneous reconstruction of discrete distributions of absorption coefficient, refractive index and a dark-field scattering coefficient. This technique avoids prior phase retrieval in the tomographic projection images and thus in principle allows reconstruction from tomographic data with less than three phase steps per projection. A numerical phantom is defined which is used to evaluate convergence of the technique with regard to photon statistics and with regard to the number of projection angles and phase steps used. It is shown that the use of a random phase sampling pattern allows the reconstruction even for the extreme case of only one single phase step per projection. The technique is successfully applied to measured tomographic data of a mouse. In future, this reconstruction technique might also be used to implement enhanced imaging models for X-ray Talbot-Lau tomography. These enhancements might be suited to correct for example beam hardening and dispersion artifacts and improve overall image quality of X-ray Talbot-Lau tomography.

## Introduction

X-ray Talbot-Lau interferometry [1–3] is a viable candidate for the implementation of a phase-contrast X-ray imaging method into laboratory environments where only low brilliance X-ray sources, i.e. X-ray tubes, are available. This includes possible applications such as non destructive testing, small animal imaging and diagnostic use in clinical environments. The method provides three different images, an absorption image, a differential phase image and a so-called dark-field image. Especially the dark-field image [4–6] has shown to be a source of information that is complementary to information that can be drawn from the absorption or differential phase images. In recent years it has also been shown that computed tomography [7–11] for all three images is possible, and that various reconstruction approaches known from absorption computed tomography can be applied to the differential phase and the dark-field image domain.

Yet, there are drawbacks of the method that might have an influence on its success for practical applications. To obtain an artifact free reconstruction of all three projection images, several repeated measurements, the so called phase steps, are needed for each projection. This has an impact on the acquisition sequence and thus time needed, especially for computed tomography. Rotation has to be stopped for each rotation angle to be able to perform all needed phase step measurements. Alternatively, several full rotations have to be performed to have repeated opportunities to obtain different phase-step measurements at the same rotation angle. There are approaches [12–16] that try to solve this but such approaches come with certain limitations or introduce errors. Another issue are redundancies within the three images, which makes it hard to extract the superimposed complimentary information, especially within the dark-field image.

Iterative reconstruction methods have already been applied successfully, for instance by [17, 18]. Methods developed in [14–16] try to reduce the total acquisition time by reducing the number of rotation steps. But all these approaches rely on the usual two step process where first projection images are reconstructed from phase stepping curves and second CT images are reconstructed from the projection images. The reconstruction ansatz by [19] and [13] work with a single step per projection. No phase stepping procedure is needed here. One phase step position is sufficient because the differential phase shift induced by the object is approximated by the linear part of a Taylor expansion of the sinoidal phase-stepping curve. Thus, the mentioned two-step reconstruction process is again followed here. But only objects with sufficiently small refractive index gradients can be reconstructed correctly. For clinical applications this restriction might be crucial.

In this work we present a reconstruction approach that simultaneously reconstructs the absorption coefficient, the refractive index and the dark-field scattering coefficient in X-ray Talbot-Lau tomography. The reconstruction is performed via a likelihood maximization and avoids an intermediate or prior retrieval of projection images of absorption, differential phase and dark-field. Thus, reconstruction is done directly from the measured phase-step projection data. This approach in principle allows a tomographic reconstruction with one single phase step position per rotation angle. This work is based on past work of the authors documented in a preprint manuscript on arXiv [20]. The main differences to this preprint concerning the method are in choosing an existing conjugate gradient implementation as maximization strategy for the likelihood instead of using a self implemented gradient ascent. The method used in this work will be described in detail in Sec. Methods of this article. Regarding the results, the major difference is that in [20], we used tomographic data that has been simulated with the help of our simulation framework for coherent X-ray imaging [21]. Instead, we will first use a numerically defined phantom using the forward projecting model of the reconstruction itself to obtain tomographic data. This allows to calculate the errors of the reconstruction and evaluate convergence of the method. Additionally, we show that with this method reconstruction of tomographic data of thorax and abdomen of a mouse obtained with an experimental apparatus is possible.

## Methods

### X-ray Talbot-Lau interferometry

**Fig 1:**
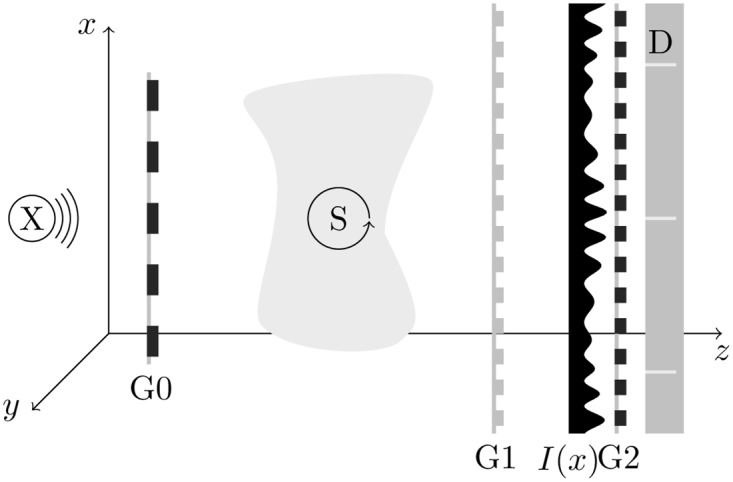
Schematic drawing of an X-ray Talbot-Lau interferometry setup. X-ray source X, source grating G0, sample S, diffraction grating G1, intensity diffraction pattern *I*(*x*), analyzer grating G2 and pixelated X-ray detector D. For tomography the sample is rotated around the y-axis as indicated.

Fig 1 shows a schematic drawing of a setup for X-ray Talbot-Lau interferometry. The Talbot-Lau interferometer, consisting of an absorbing source grating G0 and a diffraction grating G1, is illuminated by an X-ray source. Due to sufficient conditions on spatial coherence of the illumination provided by the source and the source grating, diffraction by G1 induces a fringe pattern of the intensity distribution in front of the detector. Typically, the fringe pattern is not resolvable with the detector. Thus, an absorbing analyzer grating G2, matching the spatial frequency of the fringe pattern, is placed in front of the detector.

The intensity measured in a pixel of the detector depends on the offset of the analyzer grating relative to the fringe pattern perpendicular to the grating bars. Thus, a repeated measurement of the detected intensity, with varying analyzer grating offset, provides a so called phase stepping curve with intensity *N*_*i*,*s*_ for each pixel *i* and phase step *s*. The phase stepping curve is periodic and the frequency of the first order harmonic is given by the spatial frequency of the analyzer grating. Having at least three phase steps per pixel, it is possible to reconstruct mean *m*_*i*_, amplitude *a*_*i*_ and phase *ϕ*_*i*_ of the first order harmonic for each pixel *i*. If these data are obtained for a measurement with sample, which can be placed between any two gratings, and without sample, indicated by a superscript zero, it is possible to calculate three different images. The absorption image which is given by the transmission

$$T_{i}=\frac{m_{i}}{m_{i}^{0}},$$ (1)

which is the ratio of mean values. The differential-phase image

$$\Delta \phi_{i}=\phi_{i}-\phi_{i}^{0},$$ (2)

which is given by the difference of the phase values. And, the dark-field image

$$D_{i}=\frac{V_{i}}{V_{i}^{0}},$$ (3)

which is given by the ratio of visibilities

$$V_{i}=\frac{a_{i}}{m_{i}}\;\text{and}\;V_{i}^{0}=\frac{a_{i}^{0}}{m_{i}^{0}}$$ (4)

with and without sample. Usually, the values *m*, *a* and *ϕ* are retrieved from phase stepping curves, having an equidistant sampling over one analyzer grating period, by a discrete Fourier transform.

### Tomographic imaging model

In the case of tomography with a Talbot-Lau interferometer, the sample or the setup can be rotated around an axis parallel to the fringe pattern (y-axis in Fig 1), to allow for several projection angles. In the following, the linear index *i* denotes a certain ray from source to certain pixel in the set of pixels for all projection angles. A measurement with a pixelated detector at several projection angles provides phase-step intensities *N*_*i*,*s*_ for the pixel associated with ray *i* and phase steps *s*.

#### Forward projection

In the following, we assume the sample volume being divided into discrete rectangular volume elements, indexed by *j*. Each volume element *j* has associated a scalar attenuation coefficient *μ*_*j*_, a scalar refractive index *δ*_*j*_ and a scalar scattering coefficient *σ*_*j*_. Absorption *t*_*i*_ and dark-field *d*_*i*_ values are given by

$$t_{i}=\sum_{j}M_{i,j}\mu_{j}$$ (5)

and

$$d_{i}=\sum_{j}M_{i,j}\sigma_{j}.$$ (6)

The differential phase is given by

$$\Delta \varphi_{i}=\sum_{j}\Delta M_{i,j}\delta_{j}.$$ (7)

The coefficient *M*_*i*,*j*_ is given by the length of the intersection of the ray *i* from source, which is assumed to be point-like, to pixel associated with ray *i* with the volume element *j*. The ray for projection *i* is defined by the position of the X-ray source and the center of pixel associated with ray *i*. The coefficient Δ*M*_*i*,*j*_ is given by the difference

$$\Delta M_{i,j}=M_{i,j}^{+}-M_{i,j}^{-}$$ (8)

of the coefficients $M_{i,j}^{+}$ and $M_{i,j}^{-}$. The coefficients $M_{i,j}^{\pm }$ are given by the lengths of the intersections of displaced rays for ray *i* with the volume element *j*. The displaced rays are defined by the position of the X-ray source and points that are displaced by plus or minus the pitch of the pixel associated with ray *i* relative to the center of this pixel. The displacement occurs within the plane of the detector perpendicular to the direction defined by the analyzer grating bars (x-axis in Fig 1). In this work, the coefficients *M*_*i*,*j*_, $M_{i,j}^{+}$, $M_{i,j}^{-}$ are calculated with Siddon’s method [22].

#### Expected phase sampling curve

Given the projected values *t*_*i*_, *d*_*i*_ and Δ*φ*_*i*_ depending on *μ*_*j*_, *σ*_*j*_ and *δ*_*j*_, as defined by Eqs (5)–(7), we expect a measurement given by the expected phase stepping curve

$${\bar{N}}_{i,s}={\bar{N}}_{i}\cdot \left[1+{\bar{V}}_{i}\cdot \cos\left(\varphi_{i,s}^{0}+\Delta \varphi_{i}\right)\right]$$ (9)

measured in the pixel associated with ray *i* and phase step *s*, with the expected mean

$${\bar{N}}_{i}=N_{i}^{0}\cdot T_{i}=N_{i}^{0}\cdot \exp\left(-t_{i}\right)$$ (10)

and the expected visibility [23]

$${\bar{V}}_{i}=V_{i}^{0}\cdot D_{i}=V_{i}^{0}\cdot \exp\left(-d_{i}\right).$$ (11)

$N_{i}^{0}$ is the expected mean and $V_{i}^{0}$ is the expected visibility for a measurement without sample for ray *i*. The phase step position $\varphi_{i,s}^{0}$ describes the relative phase at which the phase step *s* for ray *i* is taken. The values $N_{i}^{0}$, $V_{i}^{0}$, $\varphi_{i,s}^{0}$ can be obtained from a measurement of the phase stepping curve without sample.

### Likelihood

We assume *θ* = (*μ*_*j*_, Δ*φ*_*j*_, *σ*_*j*_) to be the vector of parameters describing the sample volume and *N* = (*N*_*i*,*s*_) to be the vector of all intensities. The likelihood [24] *L*(*θ*|*N*) of parameters *θ* given the vector of intensities *N* is then expressed by

$$L\left(\theta |N\right)=\prod_{i,s}P_{{\bar{N}}_{i,s}\left(\theta \right)}\left(N_{i,s}\right).$$ (12)

With $P_{{\bar{N}}_{i,s}\left(\theta \right)}(N_{i,s})$ being the probability of obtaining the intensity *N*_*i*,*s*_ for ray *i* and phase step *s* given that ${\bar{N}}_{i,s}\left(\theta \right)$ is expected. The expected intensity as a function of parameters *θ* is given by Eq (9). In the following, we assume that the conditional probability $P_{{\bar{N}}_{i,s}\left(\theta \right)}(N_{i,s})$ is given by a Poisson distribution

$$P_{{\bar{N}}_{i,s}\left(\theta \right)}\left(N_{i,s}\right)=\frac{{{\bar{N}}_{i,s}\left(\theta \right)}^{N_{i,s}}}{N_{i,s}!}e^{-{\bar{N}}_{i,s}}.$$ (13)

With this choice of a Poisson distribution we implicitly assume an ideal photon counting X-ray detector that has no additional noise contributions besides the photon-number noise.

### Implementation of maximum likelihood reconstruction

To reconstruct the sample volume described by *θ* from the given values *N*, the global maximum of the likelihood *L*(*θ*|*N*) as a function of parameters *θ* has to be found. It is equivalent to find the global minimum of the the negative logarithm of the likelihood

l(θ|N)=-lnL(θ|N) (14)

as a function of *θ* given intensity *N*. Thus, with Eqs (12), (13) and (14) can be expanded to

$$l\left(\theta |N\right)=\sum_{i,s}\left[-N_{i,s}\ln\left({\bar{N}}_{i,s}\left(\theta \right)\right)+\ln\left(N_{i,s}!\right)+{\bar{N}}_{i,s}\right].$$ (15)

The search for the minimum of *l*(*θ*|*N*) is performed by using the *fmin_cg* Python [25] function from the *scipy.optimize* [26] module. It uses a conjugate gradient method [27] to find a minimum for a given function. Thus, a function returning the negative log-likelihood *l*(*θ*|*N*) for given parameters was implemented. Additionally, a function returning the gradient ∇_*θ*_ *l*(*θ*|*N*) of the negative log-likelihood with respect to the parameters *θ* was implemented. The gradient function can be passed as argument to *fmin_cg*, as a replacement to the numerical gradient approximation, that would be done instead. The gradient

$$\nabla_{\theta }l\left(\theta |N\right)=\left(\frac{\partial }{\partial \mu_{j}}l\left(\theta |N\right),\;\frac{\partial }{\partial \delta_{j}}l\left(\theta |N\right),\;\frac{\partial }{\partial \sigma_{j}}l\left(\theta |N\right),\right)$$ (16)

that is returned by the implemented gradient function is given by

$$\frac{\partial }{\partial \mu_{j}}l=\sum_{i,s}\left(N_{i,s}-{\bar{N}}_{i,s}\right)\cdot M_{i,j},$$ (17)

$$\frac{\partial }{\partial \delta_{j}}l=\sum_{i,s}\left(\frac{N_{i,s}}{{\bar{N}}_{i,s}}-1\right)\cdot {\bar{N}}_{i}{\bar{V}}_{i}\cdot \sin\left(\varphi_{i,s}^{0}+\Delta \varphi_{i}\right)\cdot \Delta M_{i,j}$$ (18)

and

$$\frac{\partial }{\partial \sigma_{j}}l=\sum_{i,s}\left(\frac{N_{i,s}}{{\bar{N}}_{i,s}}-1\right)\cdot {\bar{N}}_{i}{\bar{V}}_{i}\cdot \cos\left(\varphi_{i,s}^{0}+\Delta \varphi_{i}\right)\cdot M_{i,j}$$ (19)

The implementation was done in part within pure Python and in part within an Python extension module written in C++.

### Mouse sample used in tomography

In this work tomographic raw data of a mouse sample originally acquired for [28] is reused. In the original study, the C57BL/6 adult male mouse was randomly selected from carbondioxide-killed ex-breeding stock at the Franz-Penzold-Zentrum, Friedrich-Alexander-Universität Erlangen-Nürnberg, animal facility for investigation. The animal was not killed specifically for this or the original study [28]. The animal was press fit in a 50 ml conical polyethylene tube to reduce any movement while the image acquisition was carried out. No further preparation procedures with the sample were done. An ethics approval was not required in this case and in the case of the original study. This and the original study [28] comply to the animal welfare and standard procedures of the Franz-Penzold-Zentrum and local legislation.

The images were taken with a Talbot-Lau set-up with a Siemens MEGALIX X-ray tube driven at 60 kV acceleration voltage. For photon detection, the Varian PaxScan 2520D flat panel detector with CsI as scintillation material and a pixel size of 127 x 127 *μm*^2^ was used. The gratings G0, G1 and G2 with grating periods of 23.95 *μm*, 4.37 *μm* resp. 2.40 *μm* and design energy of 25 keV were manufactured by Karlsruhe Institute of Technology (KIT) employing the LIGA method [29]. The grating G0 had a size of 5 x 5 cm^2^, the gratings G1 and G2 both had an effective area of 2 x 6 cm^2^ and were operated in the second fractional Talbot distance, resulting in distances G0-G1 of 161.2 cm and G1-G2 of 15.9 cm. The mouse was placed 10 cm in front of the phase grating G1.

## Results and Discussion

### Tomography of numerical phantom

**Fig 2:**
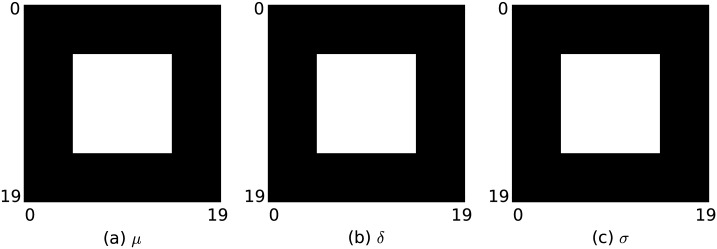
Numerical phantom. (a) Linear attenuation coefficient *μ*. (b) Refractive index *δ*. (c) Scattering coefficient *σ*. Values of the inner 10×10 region are *μ* = 0.1, *δ* = 0.75 and *σ* = 0.1. Values outside are *μ* = 0, *δ* = 0 and *σ* = 0.

For a first examination of the performance of the maximum likelihood reconstruction, a numerical phantom was designed. A two-dimensional volume is divided into 20×20 quadratic volume elements. Each volume element *j* has an edge length of 1.0 and a certain given *μ*_*j*_, *δ*_*j*_ and *σ*_*j*_ as described before. Fig 2 shows the phantom. If not stated otherwise all parameters are set to zero. A 10×10 region centered within the volume is defined, where *μ* is set to *μ*_truth_ = 0.1, *δ* is set to *δ*_truth_ = 0.75 and *σ* is set to *σ*_truth_ = 0.1. The values are chosen to obtain projected values that are on a scale that is comparable to an experimental setup.

**Fig 3:**
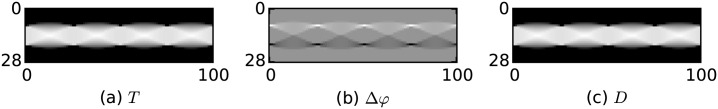
Sinogram of parallel projection of the numerical phantom onto an array of 29 pixels of pitch 1.0 for 101 rotation steps. (a) Transmission *T*. (b) Differential phase Δ*φ*. (c) Dark-field *D*.

The projection is done as described before for *R* rotation steps with angles equally distributed over an interval of 360°. The volume is parallel projected onto a pixel array with 29 pixels with a pitch of 1.0. The center of the pixel array is shifted by a quarter of the pitch from the center of the rotation axis projected onto the pixel array. Fig 3 shows the projected values of Δ*φ*_*i*_, which are found between -3.8 and 3.8, and the values of *T*_*i*_ and *D*_*i*_, which are found between 0.26 and 1.0, where *i* denotes a certain pixel for a certain projection step. For each pixel *i*, expected intensities ${\bar{N}}_{i,s}$ are created using Eq (9), with integral *s* and 0 ≤ *s* < *S*. The step phases $\varphi_{i,s}^{0}$ are given by

$$\varphi_{i,s}^{0}=\varphi_{i}^{0}+s\cdot \frac{2\pi }{S},$$ (20)

with $\varphi_{i}^{0}$ being the reference phase. From the expectation value ${\bar{N}}_{i,s}$ random values *N*_*i*,*s*_ are generated according to a poisson distribution. From this data the values $\mu_{j}^{r}$, $\delta_{j}^{r}$ and $\sigma_{j}^{r}$ are simultaneously reconstructed using the maximum likelihood approach described in Methods. A relative error of the reconstructed coefficients $c_{j}^{r}$ with *c* being either *μ*, *δ* or *σ* is given by

$$err_{c}=\frac{1}{c_{\text{truth}}}\sqrt{\sum_{j}{\left(c_{j}^{r}-c_{j}\right)}^{2}}$$ (21)

with *c*_*j*_ being the true values as defined above. The total error is given by

$$err_{\text{total}}=\sqrt{\frac{1}{3}\left(err_{\mu }^{2}+err_{\delta }^{2}+err_{\sigma }^{2}\right)}.$$ (22)

#### Convergence

In the following the iterative reconstruction is started with a volume where all parameters are set to zero. The Poisson distributed photon intensities *N*_*i*,*s*_ are created with $N_{i}^{0}=10^{12}$, $V_{i}^{0}=0.5$, $\varphi_{i}^{0}=0$, *R* = 101 and *S* = 5. The value of $N_{i}^{0}$ is chosen that photon-number noise can initially be neglected, influence of the noise level will be discussed later.

**Fig 4:**
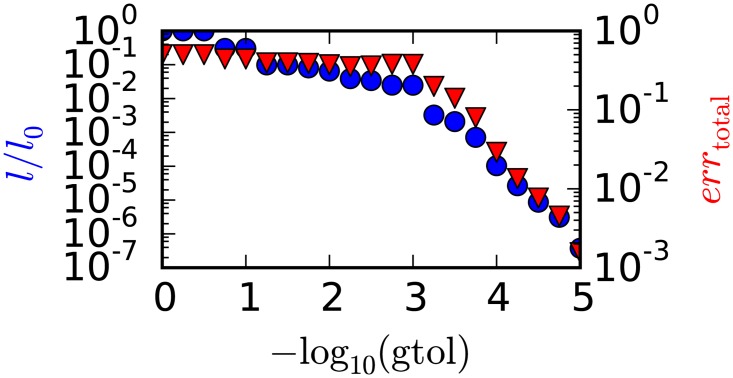
Convergence and termination. Negative logarithm of likelihood *l* and total error *err*_total_ of the reconstructed result as a function of the gradient tolerance *gtol*. *l*_0_ is the value of *l* for the starting volume parameters.

Termination of the iteration in the *fmin_cg* routine is controlled by a tolerance value for the gradient norm *gtol*. This value is per default set to 10^−5^. Fig 4 shows the negative logarithm of the Likelihood *l* and the total error *err*_total_ of the optimization result as a function of the *gtol* value. In general, a lower *gtol* value means more iterations and thus a lower value of *l*. For values of *gtol* down to 10^−3^, the total error of the reconstructed values *err*_total_ first decreases with a slight increase near 10^−3^. Below 10^−3^ the error decreases monotonically with a steeper slope than above 10^−3^, and an error in the order of 10^−3^ is reached for *gtol* = 10^−5^.

**Fig 5:**
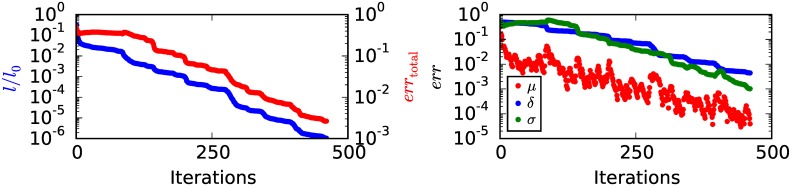
Convergence. Negative logarithm of likelihood *l*, total error *err*_total_ and the errors of *μ*, *δ* and *σ* as a function of the number of iterations with *gtol* = 10^−5^. *l*_0_ is the value of *l* for the starting volume parameters.

Fig 5 shows values of *l* and *err*_total_ after a number of iterations of reconstruction with *gtol* = 10^−5^. As has been observed before *l* decreases monotonically with the number of iterations. The total error *err*_total_ decreases within the first few iterations by about half an order of magnitude and stays at this level for about 100 iterations. Afterwards the error decreases monotonically to values in the order of 10^−3^.

Looking at Fig 5 where the errors for all three parameters are plotted, we see that the drop in the total error for the first few iterations is due to the drop of *err*_*μ*_ within the first few iterations. Afterwards the total error is dominated by the errors of *δ* and *σ*. The plateau in the total error for about 100 iterations is due to a comparable behavior of *δ* and *σ* in this region. After about 100 iterations all three errors decrease nearly monotonically, with *err*_*μ*_ staying about two orders of magnitude below the corresponding errors of *δ* and *σ*.

#### Convergence at different noise levels

**Fig 6:**
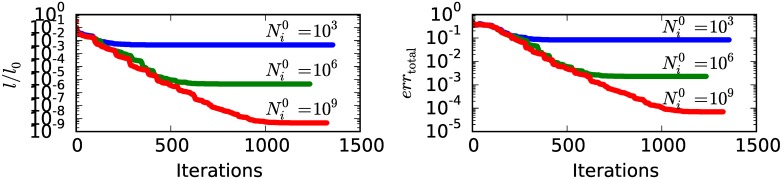
Convergence at different noise levels. (a) Negative logarithm of the Likelihood *l* and (b) the total error *err*_total_ as function of the number of iterations of the reconstruction. Evaluated for different noise levels given by the expected mean value in each pixel and projection $N_{i}^{0}$. *l*_0_ is the value of *l* for the starting volume parameters.

In Fig 6 the negative logarithm of the likelihood *l* and the total error *err*_total_ is plotted as a function of the number of iterations of the reconstruction. Random intensities *N*_*i*,*s*_ are created with $V_{i}^{0}=0.5$, $\varphi_{i}^{0}=0$, *R* = 101, *S* = 5 and three noise levels with $N_{i}^{0}$ equal to 10^3^, 10^6^ and 10^9^. The gradient tolerance *gtol* was set to 10^−8^. From Fig 6, it can be seen that *l* and *err*_total_ at first decrease with an increasing number of iterations. At a certain number of iterations, *l* and *err*_total_ begin to converge to a certain limit. This limit is lower for higher values of $N_{i}^{0}$ and thus for lower noise levels.

#### Single phase step convergence

**Fig 7:**
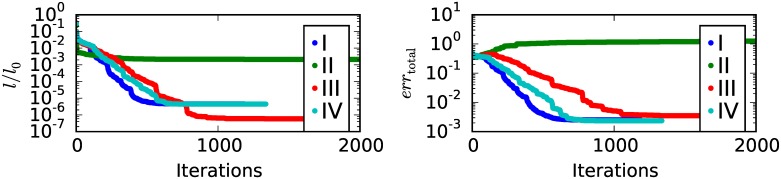
Single phase step convergence. Negative logarithm of the Likelihood *l* and the total error *err*_total_ as function of the number of iterations of the reconstruction. Evaluated for different settings regarding expected mean $N_{i}^{0}$, number of phase steps *S*, number of rotation steps *R* and reference phases $\varphi_{i}^{0}$. I: $N_{i}^{0}=1\cdot 10^{6}$, *R* = 101, *S* = 5 and $\varphi_{i}^{0}=0$. II: $N_{i}^{0}=5\cdot 10^{6}$, *R* = 101, *S* = 1 and $\varphi_{i}^{0}=0$. III: $N_{i}^{0}=5\cdot 10^{6}$, *R* = 101 and *S* = 1. $\varphi_{i}^{0}$ are constant for all pixels of one rotation step but randomly chosen from a uniform distribution between 0 and 2*π* for each rotation step. IV: $N_{i}^{0}=1\cdot 10^{6}$, *R* = 505 and *S* = 1. $\varphi_{i}^{0}$ are constant for all pixels of one rotation step but randomly chosen from a uniform distribution between 0 and 2*π* for each rotation step. Cases I to IV have the same total number of counts and thus the same total noise level. Case II has a total of 16890 iterations. Values are only shown up to 2000 iterations, but changes in the values shown for case II are not visible for numbers of iterations above 2000.

Fig 7 again shows the negative logarithm *l* of the likelihood and the total error *err*_total_ as function of the number of iterations of the reconstruction. Four cases with different settings regarding expected mean $N_{i}^{0}$, number of phase steps *S*, number of rotation steps *R* and reference phases $\varphi_{i}^{0}=0$ are compared. Case I with $N_{i}^{0}=1\cdot 10^{6}$, *R* = 101, *S* = 5 and $\varphi_{i}^{0}=0$ can be seen as a reference where five phase steps per rotation angle are used. In this case convergence is given and the minimum total error is limited by the noise level. In case II with $N_{i}^{0}=5\cdot 10^{6}$, *R* = 101, *S* = 1 and $\varphi_{i}^{0}=0$ only one phase step per rotation angle is used. To account for the reduced number of data points the expected mean is increased by a factor five to obtain the same total noise level as in case I before. In this case conventional phase retrieval would be impossible due to an insufficient number of phase steps per rotation angle. Convergence in the iterative reconstruction is not possible in this case too. To find a reason for this, we look at case III with $N_{i}^{0}=5\cdot 10^{6}$, *R* = 101 and *S* = 1, which is identical to case II but the distribution of reference phases $\varphi_{i}^{0}$. In this case the reference phases $\varphi_{i}^{0}$ are constant for one rotation angle. But, for each rotation angle the constant reference phase $\varphi_{i}^{0}$ is randomly selected from a uniform distribution from 0 to 2*π*. This effectively creates a kind of random phase stepping over all rotation angles. As we see in Fig 7 convergence for this case is possible again. Compared to case I convergence is slower and the minimal total error that is achievable is higher. In case IV with $N_{i}^{0}=1\cdot 10^{6}$, *R* = 505 and *S* = 1, the number of rotations steps is increased by a factor of five to 505 compared to 101 for the cases I to III. To get the same noise level compared to the cases I to III the expected mean is decreased by a factor of one fifth compared to case III. Convergence in case IV is still slower compared to case I but clearly improves compared to case III. And the minimal total error achievable in case IV is nearly the same as in case I.

To conclude, reconstruction with the simultaneous approach using only one phase step per rotation step seems possible if the distribution of reference phases $\varphi_{i}^{0}$ is not the same for alle pixels and rotations steps. A second observation, if the reduction in the number of phase steps is compensated by an equal increase of the rotation steps, nearly the same convergence and total error seem to be achievable.

### Tomography of ex-vivo mouse Thorax and Abdomen

So far, the performance of our iterative reconstruction method has been demonstrated with the simulated phantom only. In order to check the applicability of the method for real objects imaged with a real Talbot-Lau system we investigated data from a tomographic acquisition of the thoracic and abdominal region of a mouse [28]. For tomography, the mouse was rotated around the longitudinal axis in 601 steps distributed equally over 360°. For each rotation step eight phase steps, distributed equally over an interval with a width of 2*π*, were acquired. Reference projections, where the mouse sample is temporarily removed from the field of view, were acquired at the beginning and repeatedly after 15 subsequent rotation steps. The original dataset from [28] was taken and the detector resolution was reduced by summing repeatedly 15×15 pixels (rows and columns) in the field of view of the detector without overlap. The resulting field of view contains 20 pixels in longitudinal direction and 67 pixels perpendicular. For the reference projections, phase retrieval has been done via a fast Fourier transform. The resulting mean $m_{i}^{0}$, phase $\varphi_{i}^{0}$ and visibility $V_{i}^{0}$ are then interpolated to obtain reference values for each ray *i*.

#### Filtered back projection

**Fig 8:**
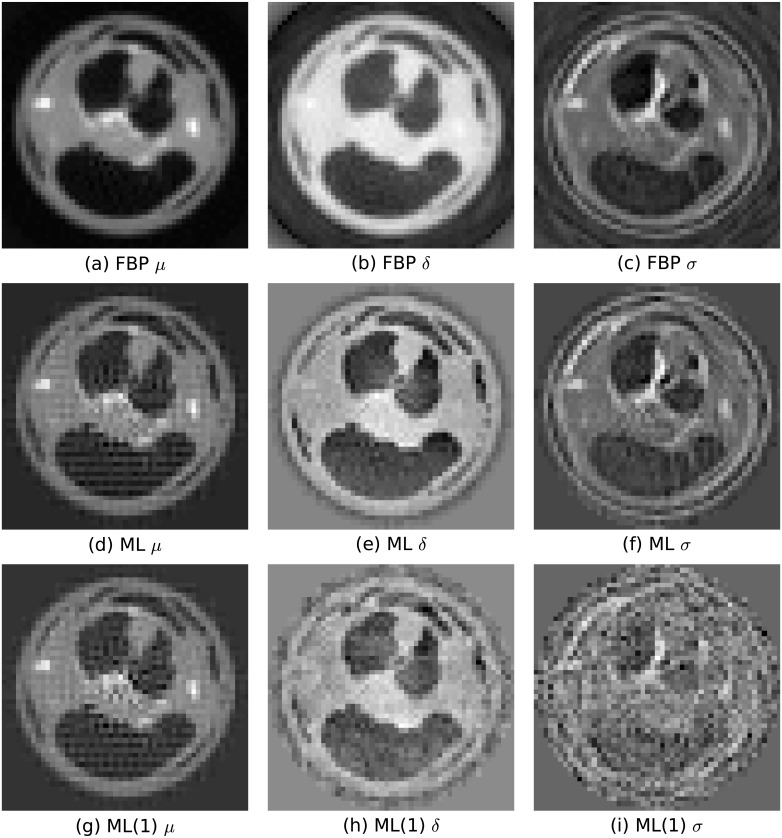
Tomographic reconstruction of a transverse plane in the region between thorax and abdomen of a mouse. 601 rotation steps around the longitudinal axis distributed over 360° with each having eight phase steps distributed over 2*π* have been acquired. (a), (d), (g): Linear attenuation coefficient *μ*, (b), (e), (h): Refractive index *δ*, (c), (f), (i): Dark-field scattering coefficient *σ*. The first row (a)–(c) shows results of a reconstruction with a filtered back projection (FBP) using a ramp filter for *μ* and *σ* and a Hilbert filter for *δ* reconstruction. The second row (d)–(f) shows results of a reconstruction with the simultaneous maximum likelihood iterative reconstruction ML. The results in the first and second row (a)–(f) are obtained from all available phase steps and projections. The third row (g)–(i) shows results obtained with the iterative approach using only 1/8 of the data, i.e. using only one phase step per rotation step (ML(1)). The phase step has been selected randomly from the available eight steps for each rotation step.

For comparison, tomographic cross sections are reconstructed with a filtered back projection (FBP). For a filtered back projection the mean *m*_*i*_, phase *φ*_*i*_ and visibility *V*_*i*_ values are retrieved for each ray *i*, again via a fast Fourier transform. From these and the reference values the negative logarithm of the transmission

$$t=-\log\left(\frac{m}{m^{0}}\right),$$ (23)

the differential Phase

$$\Delta \varphi_{i}=\varphi_{i}-\varphi_{i}^{0}$$ (24)

and the negative logarithm of the dark-field

$$d=-\log\left(\frac{V}{V^{0}}\right)$$ (25)

are calculated. The negative logarithm of transmission and dark-field are then back projected using a ramp filter, differential phase values are back projected using a Hilbert filter. Fig 8A–8C show the reconstructed *μ*, *δ* and *σ* values for one transverse plane with 51×51 volume elements. The transverse plane lies in the center of the longitudinal field of view of the detector.

#### Maximum likelihood reconstruction

For the simultaneous maximum likelihood reconstruction ML a preceding phase retrieval for each rotation step is not needed. Only the interpolated reference mean, phase and visibility values are used in the iterative reconstruction approach. Fig 8D–8F show *μ*, *δ* and *σ* values for one transverse plane with 51×51 volume elements, that are directly reconstructed from all available phase step data with the iterative method. Volume elements which are located outside a radius of 26 voxel from the center of the plane are assumed to be zero at all times. The iteration starts with all coefficients of all volume elements set to zero.

By qualitatively comparing the achieved reconstructed images we come to the following observations: The iterative reconstruction based on the same data as the filtered back projection (Fig 8D–8F) is able to converge to results that are comparable with the results provided by the filtered back projection (Fig 8A–8C). Differences in the noise and signal behavior can be observed but these might be optimized for example with additional regularization within the iterative approach. Especially, the image of the linear attenuation coefficient provided by the iterative reconstruction, shows a regular artifact pattern. This might be removed with regularization and optimized projection coefficients. For the iterative reconstruction approach a very basic algorithm is used here which offers room for improvement, nevertheless the approach in its early stage is applicable to a relevant biological sample and produces results that are comparable to those of a filtered back projection.

Considering the ingredients of the two reconstruction methods we expect that on the one hand the two-step reconstruction with phase retrieval followed by filtered back projection should provide less noise because the reconstruction of the projection images is decoupled from the CT reconstruction of the volume images. On the other hand, mechanisms like beam hardening lead to artifacts which result in correlations in the three images. A comprehensive ansatz like our iterative reconstruction addressing all three image informations simultaneously should in principle be able to better correct for such artifacts. We do not address further noise analysis here, because the trade-off between statistical and systematic image errors is a question of weighting the image information. We will cover this topic in another publication.

Fig 8G–8I, show the results of a reconstruction with the iterative method from data, where one out of the eight phase steps was randomly selected for each rotation step. This implies that the radiation dose applied is a factor of eight lower than for the images in the first and second row of Fig 8. Thus, the noise level is increased but still reasonable results can be obtained. More importantly, while one phase step does not allow to reconstruct a phase stepping curve and thus does not allow an image reconstruction for standard reconstruction methods our iterative reconstruction is still able to do so. There are approaches [12, 13] to circumvent the problem of insufficient phase step number in doing phase retrieval from phase steps of different projections. But, such approaches necessarily come with inconsistencies which the simultaneous iterative approach should be able to avoid.

## Conclusions

An analytic forward projecting model for X-ray Talbot-Lau interferometry is presented. This model calculates the expected intensities for all phase steps and projection rays for given discrete volume distributions of attenuation coefficient, refractive index and dark-field scattering coefficient. A Poisson distribution based likelihood is introduced, which quantifies the probability of the projected expectation phase-step values fitting a given measurement. In this work the likelihood is maximized using an implementation of the conjugate gradient method provided by the scipy Python package.

Using a numerical phantom we show that it is possible to simultaneously reconstruct the three volume distributions of attenuation coefficient, refractive index and dark-field scattering coefficient with the method described above, although convergence is slow and might not be guaranteed. The total error of the reconstruction depends on the noise level of the input data. Furthermore, we show that reconstruction with only one phase step per rotation step is possible if the distribution of reference phases is arranged in a suitable manner. If the reduction in the number of phase steps is compensated by an increase of the number of rotation steps convergence and achievable total error seem to be nearly equal.

Additionally, we show the tomography of a transverse plane in a region between thorax and abdomen of a mouse. Using all available phase steps we show that the reconstruction with the presented method is possible and provides results that are comparable with reconstruction results obtained via filtered back projection. Furthermore, we show that reconstruction is still possible if only one phase step per rotation step is randomly selected from the experimental data.

With the presented method reconstruction is possible for data containing only one phase step per rotation step under certain conditions. Further evaluation on the conditions for single phase step reconstruction are of interest. Additionally, the simultaneous iterative reconstruction approach might be a valuable framework for testing and improving imaging models for X-ray Talbot-Lau interferometry. Besides this aspect of modeling the physical imaging process, there is also room for improvement in numerical aspects. Different optimization strategies and last but not least regularization, which has been neglected so far, might further improve convergence and image quality. Thus, we expect advancements in quality and significance of tomographic images provided by this method in future. This might result in a more clear separation of the complementary image content among the different reconstructed images. Finally, we present a unique new and working approach for simultaneous reconstruction of attenuation coefficient, refractive index and dark-field scattering coefficient in X-ray Talbot-Lau interferometry.

## Supporting Information

S1 File Reference [20]. (PDF)

S2 File Reference [28]. (PDF)
